# Analysis of survival and prognostic factors of clear cell adenocarcinoma of the prostate: a case–control study for a rare cancer entity

**DOI:** 10.1038/s41598-023-37092-2

**Published:** 2023-06-26

**Authors:** Sijin Chen, Wei Song, Ruochen Zhang, Yiming Jin, Yan Lu, Le Lin, Liefu Ye, Tao Li, Yongbao Wei

**Affiliations:** 1 Department of Urology, Hunan Provincial People’s Hospital, The First Affiliated Hospital of Hunan Normal University, Changsha, 410005 Hunan Province China; 2 Shengli Clinical Medical College of Fujian Medical University, Fuzhou, China; 3 Department of Urology, Fujian Provincial Hospital, Fuzhou, China; 4 Department of Urology, Juntendo University School of Medicine, Tokyo, Japan

**Keywords:** Oncology, Urology

## Abstract

Clear cell adenocarcinoma of the prostate (CCPC) is a rare entity compared to acinar carcinoma of the prostate (APC). The survival rate and prognostic factors of CCPC are still unclear and deserve further study. We downloaded data on prostate cancer from the Surveillance, Epidemiology, and End Results database for 1975–2019. After inclusion and exclusion criteria, we compared APC and analyzed cancer-specific mortality (CSM) and overall mortality (OM) in CCPC patients and prognostic risk factors using a propensity score matching (PSM) study and multivariate Cox regression. We included 408,004 cases of APC as a control group and 130 cases of CCPC as a case group. Compared with APC patients, the incidence of CCPC was extremely low, and the median age of diagnosis was older (72.00 years vs. 69.00 years, *p* < 0.01). In addition, more rates were diagnosed at an earlier stage (1975–1998, 93.1% vs. 50.2%, *p* < 0.001), more unstaged or unknown stage ratios (87.7% vs. 42.7%, *p* < 0.001), and more surgical treatments (66.2% vs. 47.6%, *p* < 0.001), but the prognosis of CCPC patients was worse. After PSM, the median survival time of CCPC patients was shorter (57.50 month vs. 88.00 month, *p* < 0.01), the rate of CSM was higher (41.5% vs. 27.7%, *p* < 0.05), and the rate of OM was higher (99.2% vs. 90.8%, *p* < 0.01). In the adjusted model 2 after PSM, the CSM risk of CCPC patients reached HR 1.76 (95%CI 1.13–2.72), which was 76% higher than that of APC patients (*p* < 0.05). It was further found that surgical treatment might benefit CSM in CCPC patients (HR 0.39, 95%CI 0.18–0.82, *p* < 0.05) in Univariate analysis, but it was insignificant in further multivariate analysis. This is the first large-scale case–control report on the survival risk and prognostic factors of CCPC patients. We found that the prognosis of CCPC patients was significantly worse than that of APC. Surgery might be an effective treatment that may improve its prognosis. Clear cell adenocarcinoma, prostate, acinar carcinoma, survival rate, rare cancer, propensity score matching, case–control study.

## Introduction

Acinar adenocarcinoma (APC) is the most common type of prostate cancer (PC) according to the 2022 WHO classification^1^. APC is vital cancer that endangers men's health^2^. Proper management of these common PCs is of course critical to improve the survival status and quality of life of these patients^3^; however, it is also worth noting for the diagnosis and treatment of rare prostate cancer, such as pleomorphic giant cell carcinoma (PGCC) cases of the prostate^4^, and clear cell adenocarcinoma of the prostate (CCPC)^5^. Generally, for the scarce type of PC, such as CCPC, previous reports are reported as case reports or case series^5–9^. However, these reports provide very little valuable information in today’s evidence-based medicine; they only supply the lowest evidence level^10^. Herein, we reported, for the first time, a scarce type of PC-CCPC with the largest sample size and compared it with common APCs to improve understanding of this rare type of PC.

## Methods

### Data collection

We obtained the data usage permission of the upstaged Surveillance, Epidemiology, and End Results database in Dec. 2022 and downloaded data on prostate cancer between 1975 and 2019. Inclusion criteria were (1) all data of prostate cancer diagnosed between 1975 and 2019; (2) pathological diagnosis of APC as the control group, or CCPC as the case group. In addition, the exclusion criteria were (1) those diagnosed with CCPR but not originating from the prostate, (2) deleting the data whose survival period was less than one month, (3) deleting the data of patients younger than 18 years old and older than 90 years old, (4) deleting the data of cancer-specific mortality (CSM) with unknown cause of death. The research team keeps all data and is only used for this research.

### Statistical methods

The data we included in the study included age (years old =  ys), diagnosis year (divided into two groups according to the median, from 1975 to 1998, from 1999 to 2019), race, income, home, stage, radiation therapy, cancer-direct surgery (surgery), systemic therapy, CSM, overall mortality (OM), prostate-specific antigen (PSA), Gleason score, and survival time (month = mo). We used SPSS 27.0 (IBM, Armonk, NY, USA) for data calculation and processing. Among them, age and survival time were continuous variables, represented by median and quartile (IQR), and other data were categorical variables. The two groups were compared using the nonparametric Mann–Whitney U tests with two independent samples and the chi-square test. We next used propensity score matching (PSM) to perform baseline value matching on two groups of data, including age, year, race, marriage, income, home, stage, radiation therapy, surgery, and systemic therapy. The priority complete randomization mode was made, with the matching volume was 0.0001, and the two groups were matched according to 1:1. The hazard ratio (HR) and 95% confidence intervals (CI) of CCPC relative to APC's CSM and OM were calculated using multivariate Cox regression for the data before and after PSM. The correction models were divided into three categories, namely non-adjusted model, adjusted model 1 (adjusted for age, year of diagnosis, race, marriage, income, home, and stage), and adjusted model 2 (adjusted for age, year of diagnosis, race, marriage, income, home, and stage, radiotherapy, surgery, and systemic therapy). Then use the ggsurvplot function in the library (survminer) and library (survival) in the R studio (2022.07.01. + 554) software to make the survival curves of CSM and OM before and after PSM. Finally, we performed Univariate and Multivariate binary logistic regression analysis on the survival factors of CCPC patients, with CSM and OM as dependent variables and all other variables as independent variables. A p-value less than 0.05 was considered to have a significant statistical difference.

## Ethical approval

This study was exempt from local research ethics committee approval, considering that the Surveillance, Epidemiology, and End Results database were de-identified and publicly available for research use.

## Results

### Baseline comparison between the case and control groups

**Figure 1:**
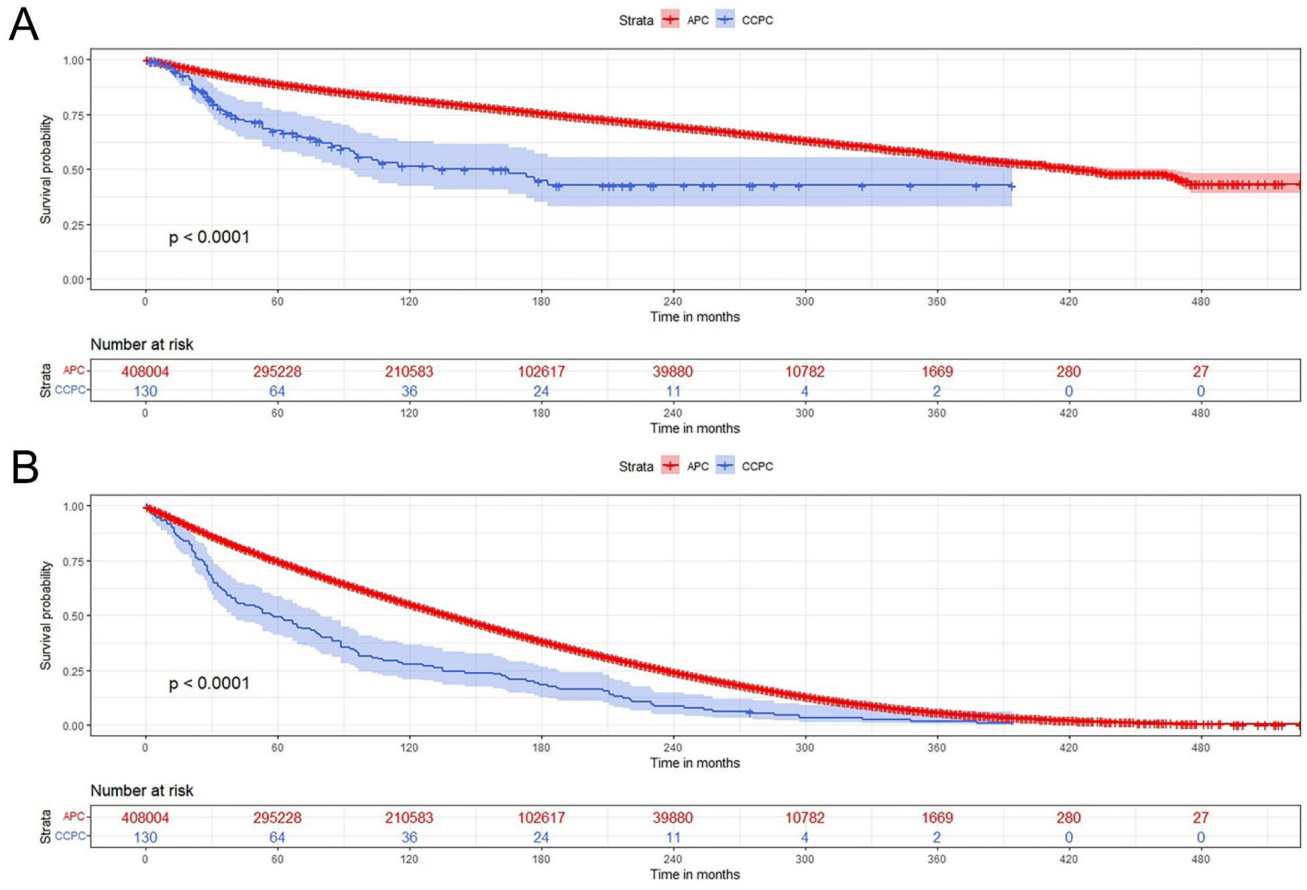
The survival rates of CCPC patients compared with those with APC. (1A) CPCC patients had a higher ratio of CSM (41.5% vs. 20.0%); (1B) CPCC patients had a higher ratio of OM (99.2% vs. 68.1%) (both *p* < 0.001). CCPC** = **Clear cell adenocarcinoma of the prostate; APC = acinar carcinoma of the prostate; CSM = cancer-specific mortality; OM = overall mortality.

We obtained 423,293 cases of APC and 138 cases of CCPC. After exclusion criteria, 408,004 cases of APC and 130 cases of CCPC were included in this study for analysis. We found that compared with APC patients, the incidence of CCPC is extremely low and rare. In addition, the median age of diagnosis of CCPC patients was older (72.00ys vs. 69.00ys, *p* < 0.01) (Table 1) (17 cases were between 46 and 60 years old; the age range was 46 to 89 years old), and more rates were in those diagnosed earlier (1975–1998, 93.1% vs. 50.2%, *p* < 0.001), had a higher proportion of unstaged or unknown stage (87.7% vs. 42.7%, *p* < 0.001), and more received surgical treatment (66.2% vs. 47.6%, *p* < 0.001). However, the prognosis of CCPC patients was worse. The median survival time was shorter (57.50mo vs. 123.00mo), among which CPCC patients had a higher ratio of CSM (41.5% vs. 20.0%) (Fig. 1A), and a higher ratio of OM (99.2% vs. 68.1%) (Fig. 1B) (all *p* < 0.001). There were no significant differences in race, marriage, income, radiation therapy, and systemic therapy between the two groups (all *p* > 0.05).

**Table 1:** bassline comparisons between the case and control groups.

	APC n = 408,004		CCPC n = 130		*P* value
	N	%	n	%	
Age (years) medium (IQR)	69.00 (63.00–76.00)		72.00 (65.00–79.00)		< 0.01
Diagnosis year					< 0.001
1975–1998	205,021	50.2	121	93.1	
1999–2019	202,983	49.8	9	6.9	
Race					0.08
White	345,440	84.7	118	90.8	
Non-white	59,772	14.6	12	9.2	
UN	2792	.7	/	/	/
Marriage					0.87
Married	288,892	70.8	99	76.2	
Single	81,674	20.0	27	20.8	
UN	37,438	9.2	4	3.1	
Income					0.07
< $75,000	170,918	41.9	21	16.2	
≥ $75,000	150,166	36.8	9	6.9	
UN	86,920	21.3	100	76.9	
Home					< 0.05
Big city	159,423	39.1	9	6.9	
Small city	155,116	38.0	20	15.4	
UN	93,465	22.9	101	77.7	
Gleason score					< 0.05
≤ 6	8512	2.1	/	/	
7–10	12,567	3.1	/	/	
TURP	3604	.9	/	/	
UN	383,321	94.0	130	100	
PSA					0.15
< 98.0 ng/ml	5468	1.3	/	/	
≥ 98.0 ng/ml	6092	1.5	/	/	
UN	396,444	97.2	130	100	
Seer-Stage					< 0.001
Localized and regional	222,304	54.5	14	10.8	
Distant	11,658	2.9	2	1.5	
Unstaged or UN	174,042	42.7	114	87.7	
Radiation					0.55
Yes	132,389	32.4	39	30.0	
No/UN	275,615	67.6	91	70.0	
Surgery					< 0.001
Yes	194,360	47.6	86	66.2	
No/UN	213,644	52.4	44	33.8	
Systemic therapy					0.94
Yes	3371	.8	1	.8	
No/UN	404,633	99.2	129	99.2	
CSM					< 0.001
Alive or other cause of death	326,355	80.0	76	58.5	
Dead	81,649	20.0	54	41.5	
OM					< 0.001
Alive	130,322	31.9	1	.8	
Dead	277,682	68.1	129	99.2	
Survival time (mo) medium (IQR)	123.00 (53.00–180.00)		57.50 (25.50–137.50)		< 0.001

CCPC** = **Clear cell adenocarcinoma of the prostate; APC = acinar carcinoma of the prostate; CSM = cancer-specific mortality; OM = overall mortality.

### Comparison between the two groups of case and control after PSM

**Figure 2:**
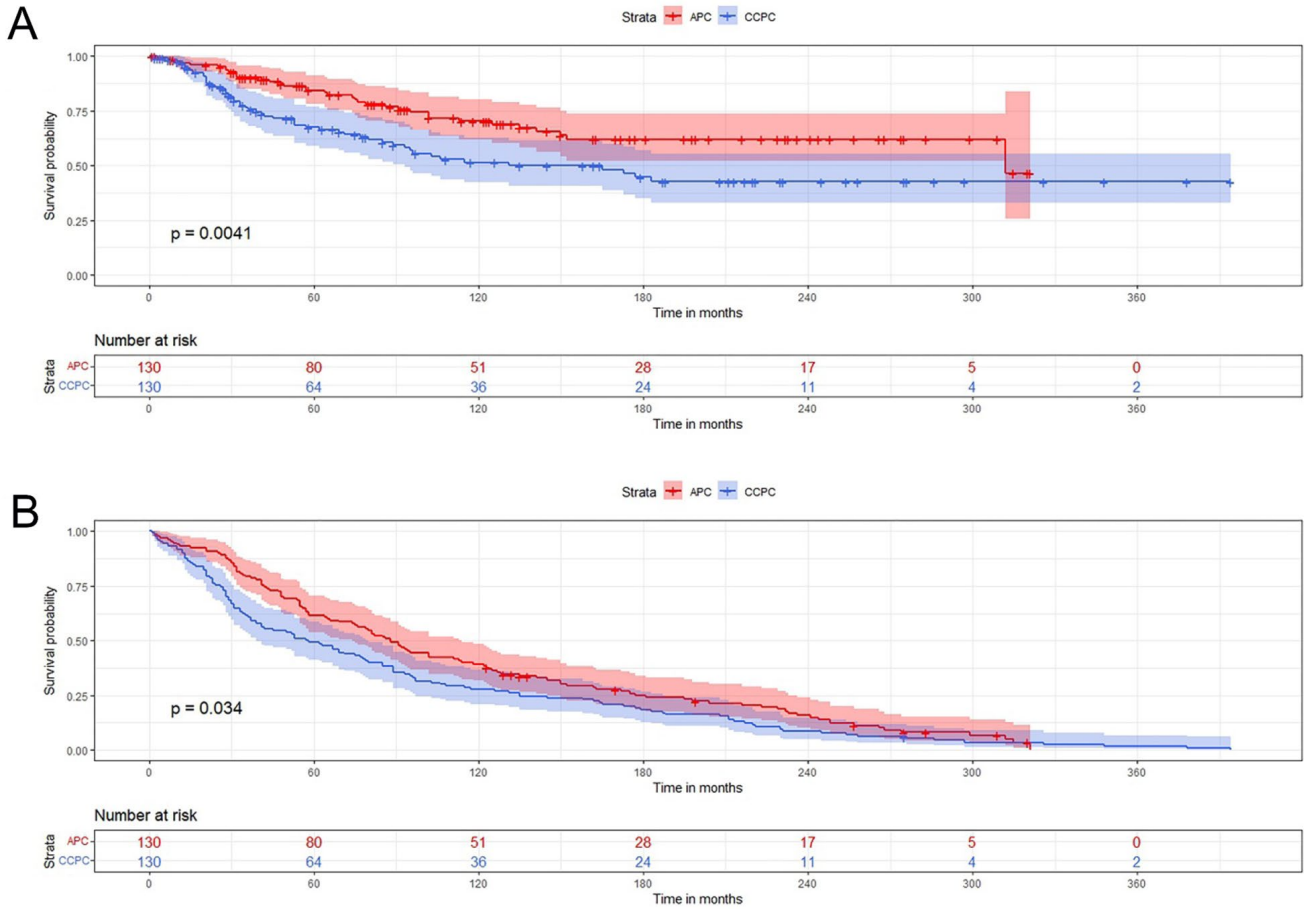
The survival rates of CCPC patients compared with those with APC after PSM. (2A) CCPC patients had a higher CSM ratio (41.5% vs. 27.7%, *p* < 0.05); (2B) CCPC patients had a higher OM ratio (99.2% vs. 90.8%, *p* < 0.01). CCPC** = **Clear cell adenocarcinoma of the prostate; APC = acinar carcinoma of the prostate; CSM = cancer-specific mortality; OM = overall mortality; PSM = propensity score matching.

After PSM, we obtained 130 patients with APC and 130 patients with CCPC. We found that the two groups of patients were perfectly matched in terms of baseline variables, including age, year, race, marriage, income, home, stage, radiation therapy, surgery, and systemic therapy, and there was no significant difference between the two groups (all *p* > 0.05) (Table 2). But the prognosis of CCPC patients was still worse, with shorter median survival (57.50mo vs. 88.00mo, *p* < 0.01), a higher CSM ratio (41.5% vs. 27.7%, *p* < 0.05) (Fig. 2A), and a higher OM ratio (99.2% vs. 90.8%, *p* < 0.01) (Fig. 2B).

**Table 2:** bassline comparisons after PSM between the case and control groups.

	APC n = 130		CCPC n = 130		P value
	n	%	n	%	
Age (years) medium (IQR)	72.00 (66.00–78.00)		72.00 (65.00–79.00)		0.89
Diagnosis year					0.14
1975–1998	114	87.7	121	93.1	
1999–2019	16	12.3	9	6.9	
Race					1.00
White	118	90.8	118	90.8	
Non-white	12	9.2	12	9.2	
UN	2792	.7	/	/	/
Marriage					0.76
Married	94	72.3	99	76.2	
Single	32	24.6	27	20.8	
UN	4	3.1	4	3.1	
Income					0.55
< $75,000	20	15.4	21	16.2	
≥ $75,000	14	10.8	9	6.9	
UN	96	73.8	100	76.9	
Home					0.22
Big city	16	12.3	9	6.9	
Small city	14	10.8	20	15.4	
UN	100	76.9	101	77.7	
Gleason score					1.00
≤ 6	/	/	/	/	
7–10	/	/	/	/	
TURP	/	/	/	/	
UN	130	100	130	100	
PSA					1.00
< 98.0 ng/ml	/	/	/	/	
≥ 98.0 ng/ml	/	/	/	/	
UN	130	100	130	100	
Seer-Stage					0.14
Localized and regional	22	16.9	14	10.8	
Distant	0	0	2	1.5	
UN	108	83.1	114	87.7	
Radiation					0.79
Yes	37	28.5	39	30.0	
No/UN	93	71.5	91	70.0	
Surgery					1.00
Yes	86	66.2	86	66.2	
No/UN	44	33.8	44	33.8	
Systemic therapy					0.32
Yes	0	0	1	.8	
No/UN	130	100.0	129	99.2	
CSM					< 0.05
Alive or other cause of death	94	72.3	76	58.5	
Dead	36	27.7	54	41.5	
OM					< 0.01
Alive	12	9.2	1	.8	
Dead	118	90.8	129	99.2	
Survival time (mo) medium (IQR)	88.00 (41.75–164.00)		57.50 (25.50–137.50)		< 0.01

CCPC** = **Clear cell adenocarcinoma of the prostate; APC = acinar carcinoma of the prostate; CSM = cancer-specific mortality; OM = overall mortality; PSM = propensity score matching.

### CPCC patients had a higher risk of cancer-related death than APC patients

We found that no matter before or after PSM, whether in the non-adjusted model, adjusted model 1, or adjusted model 2, CCPC patients had a higher risk of CSM than APC patients (Table 3) (all *p* < 0.05), among them, in the adjusted model 2 before PSM, the CSM risk of CCPC patients reached HR 2.06 (95%CI 1.11–3.83), which was 106% higher than that of APC patients (*p* < 0.05). In the adjusted model 2 after PSM, the CSM risk of CCPC patients reached HR 1.76 (95%CI 1.13–2.72), which was 76% higher than that of APC patients (*p* < 0.05).

**Table 3:** Multivariable Cox proportional-hazard models for survival outcomes in patients with CCPC compared with those with APC.

Models	HR (95% CI)	*p* value
CSM		
Non-adjusted model	2.72 (2.08–3.55)	< 0.001
Adjusted model 1	2.18 (1.17–4.05)	< 0.05
Adjusted model 2	2.06 (1.11–3.83)	< 0.05
PSM non-adjusted model	1.84 (1.20–2.80)	< 0.01
PSM adjusted model 1	1.78 (1.15–2.75)	< 0.05
PSM adjusted model 2	1.76 (1.13–2.72)	< 0.05
OM		
Non-adjusted model	1.91 (1.61–2.27)	< 0.001
Adjusted model 1	1.75 (1.20–2.56)	< 0.01
Adjusted model 2	1.79 (1.23–2.61)	< 0.01
PSM non-adjusted model	1.31 (1.02–1.69)	< 0.05
PSM adjusted model 1	1.28 (0.98–1.66)	0.07
PSM adjusted model 2	1.28 (0.99–1.67)	0.06

CCPC** = **Clear cell adenocarcinoma of the prostate; APC = acinar carcinoma of the prostate; CSM = cancer-specific mortality; OM = overall mortality; CI = confidence interval; HR = hazard ratio; PSM = propensity score matching.

Adjusted model 1: Adjusted for age, diagnosis year, race, marriage, income, home, and stage.

Adjusted model 2: Adjusted for age, diagnosis year, race, marriage, income, home, and stage, radiotherapy, surgery, and systemic therapy.

Before PSM, the risk of OM in CCPC patients was also significantly increased in adjusted model 2, reaching HR 1.79 (95%CI 1.23–2.61), which was 79% higher than that in APC patients (*p* < 0.01). However, after PSM, this increased OM was only observed in the non-adjusted model. In adjusted model 1 and model 2, the increased risk of OM in CCPC patients was insignificant (*p* > 0.05).

### Surgery may improve the survival rate of CCPC patients

We used age, year, race, marriage, income, home, stage, radiation therapy, surgery, and systemic therapy as independent variables to explore the influencing factors of CSM and OM. Finally, we found that surgical treatment as a single variable can significantly bring the benefit of CSM in CCPC patients (HR 0.39, 95%CI 0.18–0.82, *p* < 0.05) (Table 4), which has a protective effect. However, after adding age and stage, this protective effect also became insignificant in multivariate analysis. No other factors, including age and stage, significantly affected OM and CSM (all *p* > 0.05). This may be related to the fact that the sample size of CCPC patients is already large enough, but more (93.1% from 1975 to 1998) are diagnosed early, and the proportion of patients with effective staging (16/130, 12.3%) is too small, and thus no more meaningful results could be drawn.

**Table 4:** Univariate and multivariate analysis of factors affecting the survival rate of CCPC patients.

	CSM						OM					
	Univariate			Multivariate			Univariate			Multivariate		
	HR	95%CI	*p*	HR	95%CI	*p*	HR	95%CI	*p*	HR	95%CI	*p*
Age	0.99	0.96–1.03	0.71	0.96	0.86–1.08	0.48	*	*	0.97	*	*	0.99
The diagnosis year 1999–2019	1.14	0.29–4.44	0.86				*	*	0.99			
Race non-white	1.46	0.44–4.79	0.53				*	*	0.99			
Marriage single	1.43	0.61–3.36	0.41				*	*	0.99			
Income ≥ $75,000	1.00	0.19–5.24	1.00				*	*	1.00			
Home big city	0.43	0.07–2.61	0.36				*	*	1.00			
Stage Localized and regional	0.40	0.02–8.07	0.55	0.77	0.03–18.19	0.87	*	*	1.00	*	*	1.00
Radiotherapy yes	0.66	0.31–1.40	0.28				*	*	1.00			
Surgery yes	0.39	0.18–0.82	< 0.05	1.17	0.01–2.85	0.22	*	*	1.00	*	*	0.99
Systemic therapy	*	*	1.00				*	*	1.00			

*Extremum.

CCPC** = **Clear cell adenocarcinoma of the prostate; APC = acinar carcinoma of the prostate; CSM = cancer-specific mortality; OM = overall mortality; CI = confidence interval; HR = hazard ratio.

## Discussion

To our knowledge, this study is the largest sample size reported to CCPC. In this study of ours, for the first time, we compared the survival risks of CCPC patients, including CSM and OM, with common types of APC, and we found that CCPC patients were older at onset, had a worse prognosis, and shorter survival than were expected to be CSM The risk is significantly higher. Therefore, surgical treatment may be essential to improve the prognosis of CCPC.

Clear cell adenocarcinoma (CCA), different from clear cell renal cell carcinoma in the kidney, is a rare malignant tumor of the genitourinary tract; its common site is the bladder or renal pelvis, and the prostate is rarely involved; and its common metastatic sites are the lung^7,11,12^. Regarding gender differences, the proportion of CCA in women is higher than in men^7^. Until 2021, Daniel et al. found that CCA reported 247 cases (PubMed in the English literature), including 203 female and 44 male cases, with a male-to-female ratio of 4.6:1^7^. They reported 15 cases of male CCA patients, 7 cases were found in the prostate or prostatic urethra, and the remaining 5 cases were found in the bladder^7^. CCPC originating from the prostate is also known as renal-type clear cell carcinoma of the prostate^7^, based on its pathological appearance similar to clear cell renal cell carcinoma^13^. It should be noted that since primary CCPC is extremely rare when diagnosing CCPC, it is necessary to pay attention to the metastatic prostatic CCA, urothelial clear cell carcinoma, metastatic clear cell renal cell carcinoma, and other rare entities^7^. There were also reports that CCPC may merge APC^14,15^, which should be noted in pathological diagnosis. Immunohistochemical staining of prostate cancer markers may be helpful for diagnosis^15,16^.

The CCPC patients Daniel et al. reported were aged 29 to 78 years (n = 7), with a median age of 49 years^7^. In contrast, the patients we included found that the onset age of these patients was generally older, with a median age of 72 years (n = 130) and an IQR (65–79 years old) ranging from 46 to 89 years old, far older than the median onset age of APC patients (The median age of APC patients was 69 years). This may be related to the significant bias of different sample sources and sample sizes. Our conclusion supports that the median age of onset of CCPC patients is relatively older, but many patients are younger (including 17 cases between 46 and 60 years old).

CCA appears to have a generally poor prognosis. Daniel et al. summarized the literature and found that the 1-year survival rate of CCA patients (n = 135) was 60.4%, 33.3% at 2 years, and 19.4% at 3 years^7^. In addition, 15 patients reported they underwent cystectomy, cystoprostatectomy, radical prostatectomy, combined or not combined with lymph node dissection, and subsequent therapies, such as radiation therapy, chemotherapy, or immunotherapy; however, the prognosis is still poor^7^. The CCPC patients we included also underwent surgery, radiotherapy, or systemic treatment, and we found that the prognosis was worse than that of APC patients. By the end of the follow-up, compared with APC matched in age, stage, and treatment, the median survival time of CCPC patients was shorter (57 months vs. 88 months), and the ratios of CSM and OM were higher. After PSM matching and adjustment (adjusted model 2), CCPC, a rare pathological type of prostate, has a 76% increased risk of CSM compared with APC but no significant increase in the risk of OM death. Using univariate and multivariate analysis, their study found that age and distant metastasis indicate a worse prognosis^7^. Interestingly, we only found that surgical treatment had a protective effect on CSM in univariate analysis, and no significant factors affecting OM were found. This may be related to the characteristics of our sample because the stage of more than 70% of CCPC patients is unknown, and the proportion of radiotherapy and systemic treatment is low. Combined with previous studies, age, stage, and treatment should have a meaningful impact on the survival and prognosis of such patients.

This study had some limitations. As mentioned above, although the sample size of our included CCPC patients was already significant, the vast majority were diagnosed between 1975 and 1998. Most patients lacked effective staging, so more meaningful results could not be obtained. It also made our research conclusions had a particular deviation. In addition, as a retrospective study, we only included part of the data in North America covered by the SEER database, so our conclusions could have been more extensive. Furthermore, the basic health status of these patients, which may contribute to the survival rate, needed to be analyzed but it was not available in the database. Our study found that the surgical treatment was insignificant in multivariate analysis after adding age and stage. No other factors, including age and stage, were supposed to be two potential critical factors that significantly affected OM and CSM in many cancer entities. Still, our study did not identify any significant prognostic risk factors for CCPC beyond surgical treatment as a single variable. In our opinion, potential reasons to interpret these results might be due to a relatively small number of CCPC cases, these cases came from a relatively large period, and even many of them were diagnosed between 1975 and 1998, during which medical care was relatively backward especially the oncological surgical outcomes. Furthermore, some other important parameters that may affect the survival rate of CCPC patients were missing in our study, including tumor size, tumor stage, lymph node involvement, distant metastasis or not, and free surgical margin or not, as they were not well recorded in the current form of the SEER database. However, as in many rare PC studies, our research might still bring specific reference significance to medical care providers and patients.

## Conclusions

This is the first time a large sample size has been reported on the survival risk and prognostic factors of CCPC patients. We compared this rare prostate malignancy with APC and found that the prognosis of CCPC patients was significantly worse than that of APC, especially since the risk of CSM was significantly increased. Surgery might be an effective treatment modality that might improve its prognosis.

## Data Availability

The datasets generated for this study are available to the corresponding author upon request.
